# Assessment of ambulance interventions; proposal of a performance measurement framework for healthcare improvement in EMS response to patient collapse

**DOI:** 10.1186/s12873-025-01206-y

**Published:** 2025-04-12

**Authors:** Kamran Idris, Zainab Mubeen, Zeeshan Noor Shaikh, Aswad Latif, Shaheryar Hasan, Arshia Khan

**Affiliations:** Department of Research, Development and Education, Sindh Integrated Emergency and Health Services, Karachi, Pakistan

**Keywords:** Collapse in ambulance, Healthcare improvement, Performance measurement, Prehospital care

## Abstract

**Background:**

Healthcare improvement at all levels involves systematic and continuous assessment of the system’s operations, efficiency, and effectiveness to ensure quality care. Likewise, in Emergency Medical Services; performance measurement and root cause analysis may aid in identifying the system inadequacies and address potential shortcomings by developing Key Performance Indicators. In this paper, we propose a tailored framework to supplement the performance measurement and healthcare improvement, primarily to monitor the quality of EMS operations and personnel for ambulance transfers, which results in patient collapses in ambulances.

**Methods:**

We developed a Performance Measurement Framework (PMF) based on three essential domains– Structure/System, Process, and Outcome. Each domain was further assigned with different KPIs to assess the performance of EMS operations and personnel during patient transfers. The framework was pilot-tested for one year from January to December 2023, where its use was limited to the cases of patient collapse in ambulances, also referred to as out-of-hospital deaths. To assess progress, we compared the incidence of CIA between the pre-implementation and post-implementation phases, with service operational metrics including coverage, fleet size, workforce, and response times.

**Result:**

Using the PMF as a tool for quality improvement, we observed a 7% reduction in the incidence of patient collapse in ambulances and a 16% reduction in life-threatening cases resulting in CIA despite increases in service coverage (37%), ambulance workforce (32%), fleet (26%), and routine interventions (11%). A slight increases in response times indicate the greater service demands. Through pilot testing, we identified operational gaps including behavioral and communication issues, adherence to SOPs, and equipment management.

**Conclusion:**

Overall, this paper proposes a performance measurement tool in the field of prehospital care for organizations to thoroughly assess and advance their operations toward healthcare improvement. The study highlights areas requiring improvement such as training guidelines, adherence to operating protocols, and resource optimization. In addition; the integration of technology and advanced training programs for the ambulance workforce may strengthen the overall EMS performance; thereby promising positive patient outcomes, and efficient service delivery and utilization.

**Trial registration:**

Not applicable.

## Introduction

Healthcare improvement refers to the systematic reassessment approach to monitor the health system’s performance, efficiency, and effectiveness in delivering quality healthcare that meets expectations [1]. In Emergency Medical Services (EMS), the concept of healthcare improvement aligns with six major principles defined by the Institute of Medicine (IOM) including patient safety, service effectiveness, patient-centered care, timeliness of care, efficiency, and equitable care [2]. Successful implementation of these principles in EMS organizations providing prehospital care in multiple countries resulted in identifying areas for future development, improved clinical performance of paramedics, and adherence to treatment guidelines while effectively contributing to overall system advancement [3–5].

Within the health system, healthcare improvement involves a series of activities that include performance measurement of services and root cause analysis of system inadequacies from different dimensions, aiding in developing an action plan to address potential shortcomings. In the context of prehospital care, the literature suggests the selection of context-specific patient-centered indicators such as on-scene time or response time intervals, administration of aspirin in acute coronary syndromes, early defibrillation, and CPR in the events of out-of-hospital cardiac arrest (OHCA) and other clinical parameters [6, 7]. The data sources for implementing improvement mechanisms involve ambulance dispatch information, patient care documents, system dashboards, and follow-up hospital records. Considering the involvement of multiple layers of EMS in delivering pre-hospital care, it is essential to employ all available data sources to identify the best possible approach for addressing quality issues [8, 9].

At both the national and international levels, there is a paucity of literature on performance measurement and healthcare improvement at the prehospital stage, for routine interventions as well as for critical cases resulting in cardiac arrest or death during patient transfer [10]. Despite the global emphasis on quality improvement in health services, performance measurement at the prehospital level is rare, especially in low-middle-income countries regardless of the type of cases owing to fragmented systems and resource limitations [11]. Whereas, in developed countries, performance evaluations of EMS are mostly centered on response times; which alone is insufficient to comprehensively represent the quality-of-service delivery and workforce compliance to SOPs [12]. Given this gap, a structured approach is necessary for the performance measurement of EMS operations and personnel, to ensure continued efforts for healthcare improvement, efficient patient management, and service delivery. Considering this scarcity, the provincial non-profit EMS organization in Sindh, Pakistan routinely analyzes ambulance interventions to monitor system performance and inform policy formulation by addressing gaps in structure, process, and outcomes.

Based on such an initiative and literature gap, we propose a tailored framework to supplement the performance measurement and healthcare improvement primarily to monitor the EMS operations and personnel. We are the first organization to develop Key Performance Indicators (KPIs) with the aim of healthcare improvement at the prehospital level; however, considering the limited resources and manual analytical approach, we restrict the application of the Performance Measurement Framework (PMF) to ambulance transfers resulting in a patient collapse in an ambulance (CIA). In this paper, CIA is a novel terminology coined by the study team that aligns with the concept of out-of-hospital patient deaths, referred to as sudden deterioration of physiological functioning witnessed by paramedics in the ambulance with the patient not exhibiting apparent signs of life and subsequently transferred to the health facility for confirmation.

The purpose of this study is to design and implement a Performance Measurement Framework (PMF) based on KPIs to assess EMS performance, improve patient management during transfers, and ensure workforce compliance with SOPs, thereby contributing to healthcare improvement and policy revisions at the organization level. For future studies, this framework may serve as a basis for EMS organizations to conduct a thorough analysis of operational dynamics in the events of the CIA, or other major emergency incidents. Our anticipated outcomes for this study include identifying areas for healthcare improvement at the prehospital level, improving patient survival rates, and overall quality of EMS organization. Following the development of PMF, we conducted pilot testing and a retrospective analysis to determine its effectiveness.

## Methods

### Design

This study was conducted in a provincially representative EMS organization in Sindh, Pakistan using a three-phased approach: (i) Development of PMF (ii) Implementation of PMF, and (iii) Impact Assessment.

#### Phase I: Development of Performance Measurement Framework (PMF)

The framework is based on three essential domains including Structure/System, Process, and Outcome. The performance of the involved actors is measured through a set of KPIs; each assigned a different weightage. The domains of PMF include:

- ***Structure/System***: Standard Operating Procedures, Infrastructure, Equipment, and Health Workforce.
- ***Process***: Steps in patient care intended to improve the patient’s condition including treatment protocols, medical administration, or patient transfer to the desired facilities.
- ***Outcome***: Changes in patient health during the transfer such as survival, morbidity and mortality, patient improvement, or satisfaction.

The framework is applicable for assessing the performance of all actors engaged from the initial call receipt to patient transfer and is applicable across all ambulance interventions. However, due to the resource constraints and time limitations to study all cases manually, we recommend the application of this framework to evaluate EMS performance specifically in the events of patient collapse in ambulances or deaths. This approach attempts to identify development areas in operations and facilitate continuous service delivery. The KPIs and their respective descriptions along with measures of verification are presented in Table 1 - Domains and KPIs of Performance Measurement Framework (PMF).

**Table 1:** Domains and KPIs of Performance Measurement Framework (PMF)

Domain	Actor	Key performance indicator (KPI)	Weightage	Definition	Measure of verification (MOVs)
Structure	EMD	EMD Compliance	10%	Compliance with the SOPs related to call-taking proficiency, emotional content, coding accuracy, dispatch process, instructions, and customer service.	Review Report by EMD-Q using AQUA Ascent 7
	ETC	ETC Compliance	5%	Compliance with SOPs related to communication skills and procedural accuracy	Call Recordings
	EMD	Call Dispatch Time	5%	The time interval from the receipt of an emergency call by EMD to the subsequent dissemination of information to ETC.	HES Portal
	Station Supervisor	Management of fleet/biomedical equipment and logistics	10%	Administration, Coordination, and Optimization of ambulance vehicles	Ambulance Response Form
		Station Supervisor Remarks **	NA	SS comments related to identified issues and mitigation strategies, if applicable	Ambulance Response Form
		Post-intervention procedures **	NA	Disinfection measures taken following the intervention/transfer	Ambulance Response Form
Process	Ambulance Crew	Movement Response Time	5%	Time elapsed from the moment the ambulance crew was notified until they initiated a movement	HES Portal
	EMT	Hospital Handling Taking	10%	Transferring responsibility, clinical information, and patient care from attendants to EMTs during home-to-ambulance transfers or from EMTs to physicians/senior staff during transfers from ambulance to health facility	Care Transfer Form
		Patient Counselling	5%	Counseling of attendants/patients to administer required treatments, medications, or procedures	Discussions with EMTs and Patient Attendants
		Patient Management	30%	Management of patient conditions in ambulance	Ambulance Response Form and Discussions with EMTs and Patient Attendants
		Documentation	NA	Maintaining documentation as per SOPs	Ambulance Response Form, Critical Transfer Form, Care Transfer Form
		Request for MO Supervision	10%	Requesting clinical assistance from on-duty Medical Officer (teleconsultation)	Ambulance Response Form
	**Medical Officer**	MO Compliance	10%	Compliance of MOs for patient management	Call Recordings
Outcome	**EMS**	Patient/Attendant Satisfaction	NA	Level of attendant satisfaction with organization service and ambulance crew performance	Call Recordings

### Scoring method

The progress against each indicator is measured using a Likert scale ranging from 0 to 3: 0 (below expectations), 1 (partially meets expectations), 2 (meets expectations), and 3 (exceeds expectations). Depending on the score, the compliance and performance are marked as 0%, 50%, 75%, and 100%, respectively. The weighted score is calculated by multiplying the obtained score with the weightage. The validation process was based on expert review, pilot testing of framework for one year, and retrospective analysis of data to ensure its reliability in assessing progress against KPIs. The scoring criteria are further presented in detail in Table 2 - Performance Measurement Framework for Collapse in Ambulance – CIA.

**Table 2:** Performance Measurement Framework for collapse in ambulance – CIA

Category	Indicator	Likert scale (0–3) – Scoring out of 100			
		Below expectations (0) 0%	Partially meet expectations (1) 50%	Meet expectations (2) 75%	Exceed expectations (3) 100%
Structure	EMD Compliance (Call Evaluation)	Non-Compliance/Low Compliant	Partially Compliant	Compliant	High Compliant
	ETC Compliance (Call Evaluation)	Non-Compliance/Low Compliant	Partially Compliant	Compliant	High Compliant
	Call Dispatch Time	More than 90 s	-		Less than 90 s
	Management of fleet/biomedical equipment and logistics	Ambulance not ready for an emergency	Real time issues	No fleet/logistic/biomedical issues	The real-time issue was killed efficiently without compromising the patient
	Station Supervisor (SS) Remarks **	No intervention of SS (Lack of signature plus remarks)	lack of SS remarks (Only signature present)	SS remarks, properly signed & errors highlighted	SS ME + Action points
	Post-intervention procedures **	Post-intervention procedure not done	-		Post-intervention procedure done
Process	Movement Response Time	Moved after 2 min	Moved within 2 min	Moved within 1 min	Movement below or equal to 30 s/staff himself intervened for emergency response
	Hospital Handing-Taking	Not done	Vitals taken and handling done	1 + checked IV-line, infusion Status, and documentation	2 + Anticipated management asked for/ done
	Patient Counseling	Not Attempted	Partially done	Done properly	Done properly/ Decision of attendant changed
	Patient Management	Not done	Partially done	Done Properly as per protocol	2 + Patient revert ROCS achieved
	Documentation	Not done/False/fake documentation	Missed some points/overwriting	Adequate documentation	Documented everything, no error, no overwriting, properly readable and matching with the treatment given
	Request for Physician supervision	Not asked or asked after 30 min	-		Asked within 15 min
	Medical Help by MO (Call Evaluation)	Medical help not provided adequately	Medical help only as per symptoms/sign	History/vitals taken, assessment done and help provided remains on call if needed, CPR protocol and dose if needed	ME + Proactive assessment and instructions
Outcome	Caller Feedback	unsatisfied	Partially satisfied.	satisfied	highly satisfied

#### Phase 2 – Implementation of Performance Measurement Framework

From January to December 2023, we implemented the PMF as a pilot test to determine its effectiveness and feasibility in highlighting systemic weaknesses for ambulance transfer cases that resulted in CIA from January to December 2023. Before implementation, a one-week training was conducted for Medical Officers (MOs) involved in conducting analysis. The training established an overall understanding of the framework, reviewed relevant procedures across all levels, and engaged participants in case-study-based discussions. Further, it has put a lot of emphasis on feedback and reporting techniques, ensuring that the MOs are equipped to apply the framework in their analysis and contribute towards improving the quality of patient care during the process of ambulance transfers. At the end of each analysis, a final report is compiled with detailed information on the patients, ambulance crew, treatments administered during transfer, attendant feedback, and completed PMF. Identified deficiencies and problems in given sections are to be escalated to the responsible departments as well so that targeted measures are proposed for implementation to prevent similar errors from happening again. At the end of every month, the senior leadership would review all reported cases in the organization with departmental representatives. This review is interpreted to provide identified issues, assign accountability to appropriate personnel, and formulate action plans for addressing performance deficiencies among involved actors. It is worthwhile to mention that the performance measurement may serve as a major incentive for EMS personnel, as it will help them to have the motivation to comply with SOPs and achieve set standards.

#### Phase 3 – Impact assessment

At the end of the pilot run for one year, we conducted a retrospective analysis using organizational records to assess the progress against service targets and operational capacities.

### Study duration

The PMF was developed over four months from August – December 2022 to identify areas needing improvement in terms of patient survival, service quality, and prevention of prehospital mortalities in ambulances during transfer. It was conceptualized and designed by a team of medical doctors from the Department of Research, Development, and Education, collaborating with other departments involved in day-to-day EMS operations. The ambulance records were retrospectively studied for three months from July to September 2024. In this study, we considered records from Jan-Dec 2022 as the pre-implementation phase while ambulance records ranging from Jan-Dec 2023 were classified as the post-implementation phase to compare the trends and study the impact of implementation.

### Study setting

The Performance Management Framework was pilot-tested on CIA cases occurring within one year.

### Sampling

Using a purposive sampling methodology, we selected ambulance records from the EMS in which a patient collapse occurred within an ambulance during January – December 2022 and January – December 2023 as pre-implementation and post-implementation phases, respectively. Data was extracted using the ambulance response forms, critical transfer forms, and care transfer forms of ambulance transfers that resulted in CIA, to ensure complete coverage of KPIs.

### Data collection

To conduct a comparative analysis, the data was collected from the pre-implementation (January-December 2022) and post-implementation phases (January-December 2023) consisting of service coverage indicators including the number of districts covered, total fleet size, ambulance crew, number of transfers, call dispatch time, response time, types of cases and their respective dispatch and response time, and patient outcomes.

### Data analysis

The data extracted from the records kept by the organizations for the two periods underwent a descriptive analysis with MS Excel v.2021 comparing pre and post-implementation records. The mean and frequencies of each of the service indicators were determined to identify improvements in EMS performance such as service delivery, reduction in the incidence of CIA, improvement in response times, compliance and adherence to SOPs, and workforce performance in relation to service coverage across both periods.

## Results

**Fig. 1:**
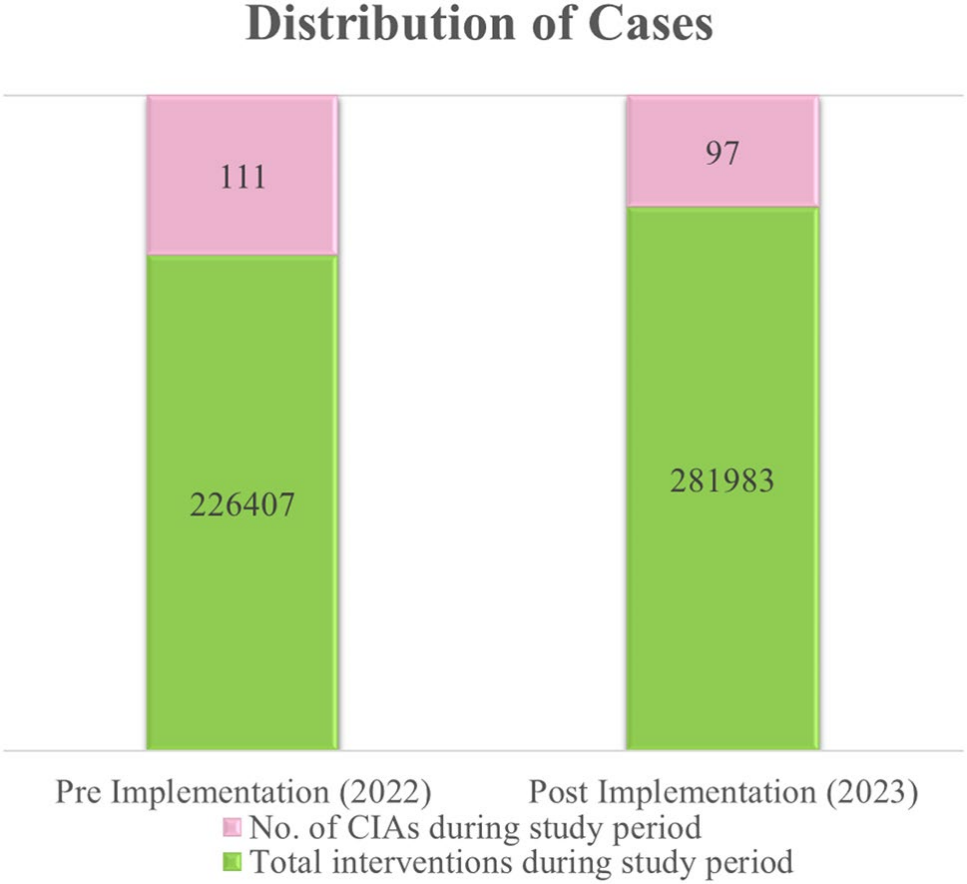
Distribution of cases in pre- and post-implementation

**Fig. 2:**
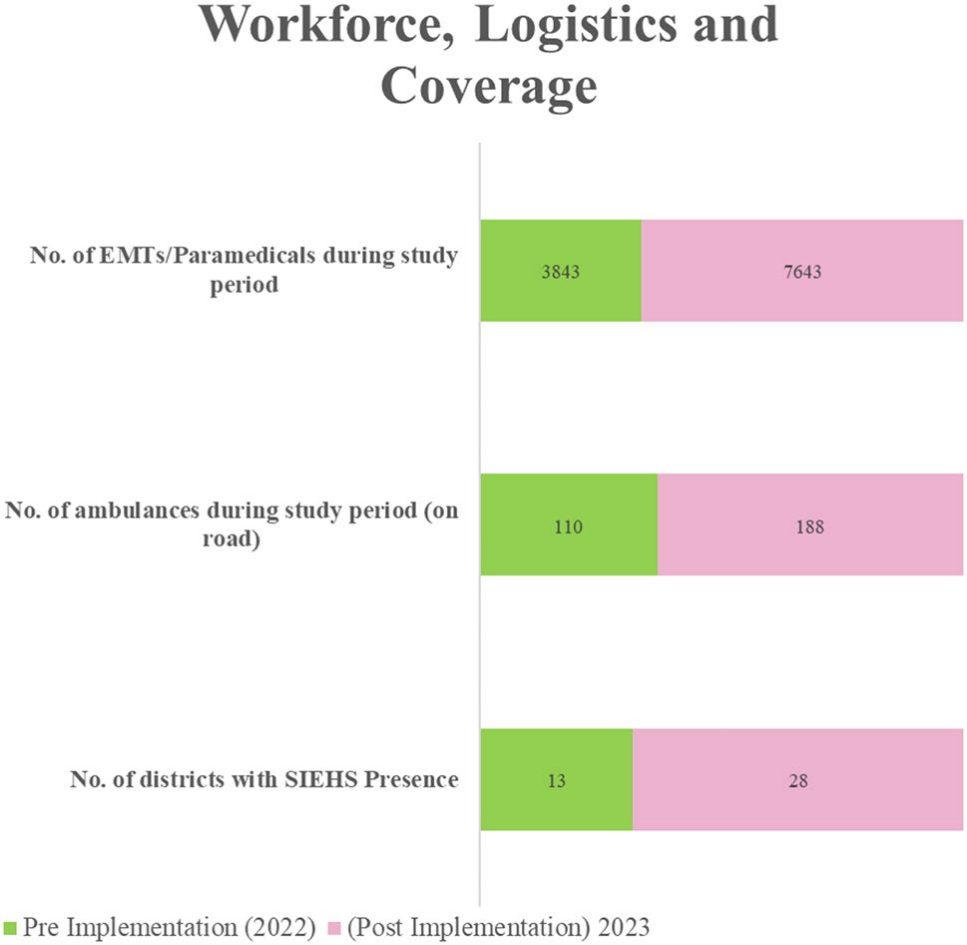
Workforce, logistics, and coverage comparison pre- and post-implementation

**Fig. 3:**
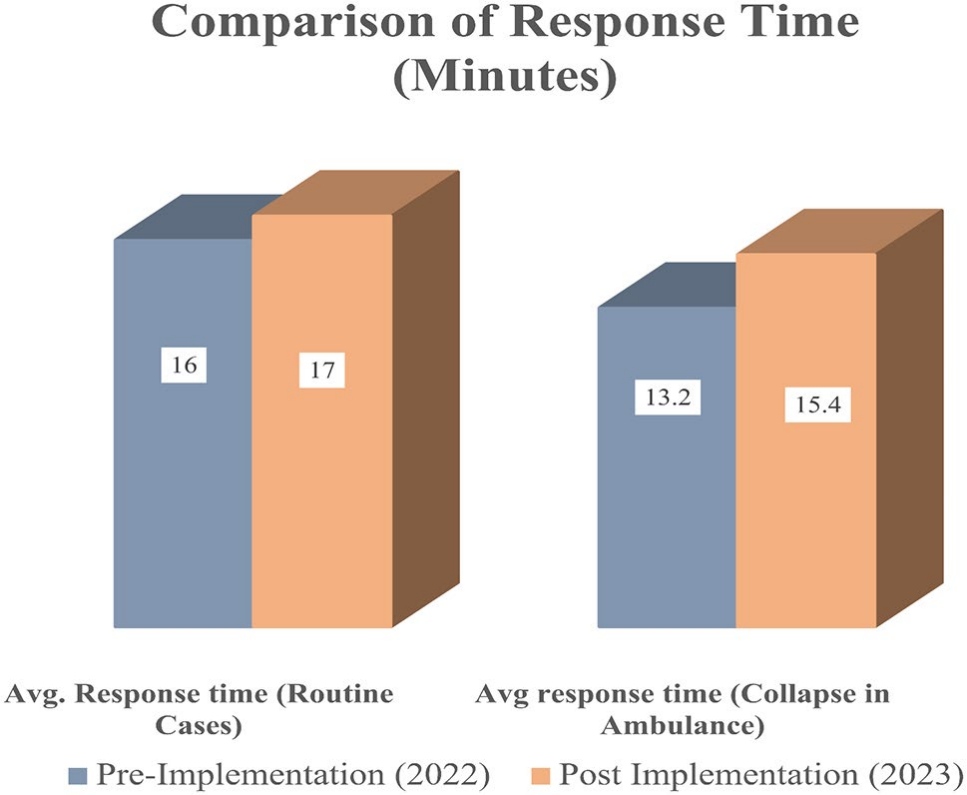
Comparison of response time (minutes) pre- and post-implementation

The key operational parameters in this study were compared between the pre-implementation (2022) and the post-implementation phase (2023) focusing on service coverage, available ambulance vehicles, and involved workforce. In addition, the average number of routine interventions performed and the average response times were assessed in both phases. Over time, the service coverage increased by 37% (2022: *n* = 13; 2023: *n* = 28) while the proportion of ambulance vehicles available on the road increased by 26% from 110 vs. >188 ambulances in a year. Similarly, the EMS workforce expanded by approximately 32%, from 3,843 EMTs and paramedics to 7,463, and routine intervention volumes by 11%, from 226,407 to 281,983 interventions. Such expansion in service operations led to a minor increase in response time from 16 to 17 min for routine interventions, as a result of a surge in demand. In terms of the occurrence of patient collapse in ambulances, the continuous efforts towards health improvement reduced by 7% from 111 to 97 cases in a period of one year. The distribution of cases, service expansions, and comparison of response times are presented in Figs. 1, 2 and 3 respectively.

**Fig. 4:**
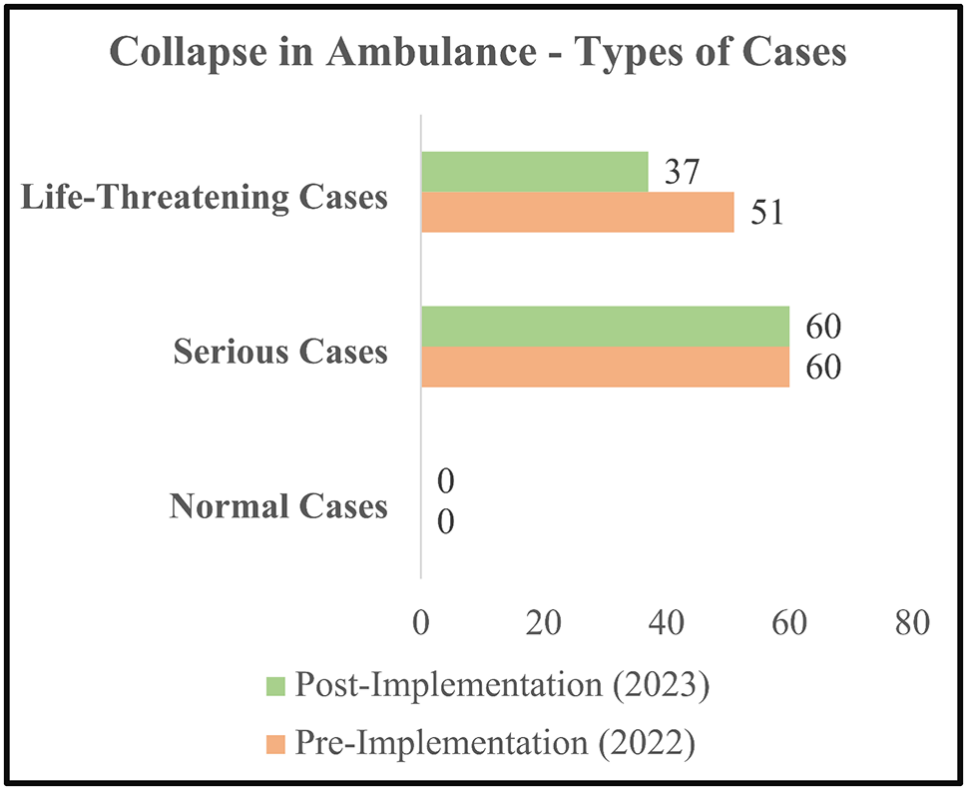
Collapse in ambulance – types of cases (pre- and post-implementation)

It is worth highlighting that no normal cases resulted in CIA, precluding any associated response time data for such cases. Whereas, the number of serious cases in both the pre-implementation and post-implementation phases resulting in CIA remained constant at 60. The analysis also reported a 16% decline in the frequency of life-threatening cases resulting in CIA from 51 to 37 cases. The average response time in CIA cases experienced a slight increase of 8% from 13.2 to 15.4 min owing to extensive distances to emergencies, unavailability of ambulances at the nearest location, and an increase in demand. Figure 4 presents the types of cases that resulted in CIA from both phases. The summary of study findings is presented in a detailed manner in Table 3 - Progress Against Service Indicators in Pre-and Post-Implementation Phases.

**Table 3:** Progress against service indicators in pre-and post-implementation phases

Service indicators	Progress		
	Pre-implementation (2022)	Post-implementation (2023)	Percentage variation (%)
**Routine Interventions**			
No. of districts with SIEHS Presence	13	28	37%
No. of ambulances during the study period (on-road)	110	188	26%
No. of EMTs/Paramedical during the study period	3843	7463	32%
Total interventions during the study period	226407.0	281,983	11%
Avg. Response time (minutes)	16	17	3%
**Collapse in Ambulance**			
No. of CIAs during the study period	111	97	-7%
Avg. Response time (minutes)	13.2	17	12%
No. of normal cases	0	0	
Avg response time of normal cases	0	0	
No. of serious cases resulted in CIA	60.0	60	0%
Avg. Response time of serious cases	12	15	12%
No. of Life-threatening cases	51	37	-16%
Avg. Response time of life-threatening cases	13.9	16	7%

Note: A value with a minus sign shows a reduction

In addition to descriptive data, the desk review of CIA reports highlighted areas that can be concentrated for better patient management in the near future. These were related to communication and behavior incompetence, incomplete adherence to dispatch protocols, minor procedural and knowledge gaps, equipment handling errors, documentation errors, and delayed decision-making. Based on the identified areas during pilot testing and retrospective analysis, we took action to address systematic challenges and improve the delivery of prehospital emergency care through the below activities:

1. Continued Medical Education (CME) – The workforce involved in CIA events were considered for refresher training focused on clinical skills, standard operating procedures (SOPs), and measures to respond proactively to the stressful circumstances experienced during patient transfer. All the education for the EMS workforce is aligned with fundamental ethical principles including patient autonomy, maximum benefit for patients (beneficence), do no harm (non-maleficence), and equality in service provision (justice).
2. Counselling Session – To assure adherence to IAED and organizational standards, we carried out individual counseling sessions for EMDs performance improvement, quality in patient care, and reduction in operational errors during intervention.
3. Biomedical Equipment Maintenance – A proactive approach was adopted to conduct monthly equipment checks in all EMS stations for functionality and preparedness. This is necessary to prevent equipment failure before critical interventions.
4. Feedback Loops: We also implemented an improved feedback mechanism to encourage continuous healthcare improvement through performance reviews, case debriefings, and patient feedback. Data collected through these measures will be used to inform broader policy change that is oriented towards the efficiency of operations and outcomes for ambulance transfers.
5. Knowledge Assessment and Continuous Learning: A fully developed knowledge assessment program was proposed to assess the competency of EMS personnel. These include regular knowledge and skill assessments by the RDE and MEAL teams related to medical knowledge, adherence to protocols, and emergency response capabilities. These assessments influence the type of ongoing training programs and professional development activities that are undertaken to continue high standards in patient care, as well as readiness for various emergency scenarios.

Briefly, through implementing these strategic actions with an iterative analysis of CIA events using the Performance Measurement Framework, we aim to propose continued healthcare improvement practices in the EMS organizations, particularly in settings where resources are limited and the burden on the healthcare system is higher. Moreover, we also aim to build upon the success of the PMF to enhance the capability of EMS to provide timely, effective, and patient-centered pre-hospital care. Such schemes demonstrate the organization’s commitment to improvement in the healthcare system and readiness for dealing with changes in population demand.

## Discussion

Given the emerging role of emergency medicine in healthcare research and global priority to the quality and safety of clinical care at the prehospital level; we proposed a framework specifically to be employed in the cases of patient collapse or deaths in ambulances. The KPIs were developed with the mutual collaboration of key stakeholders involved in the management and assessment of operations, around the EMS structure, process, and patient outcomes involving cases of CIA. Our study creates a foundation for healthcare improvement through the development of a Performance Measurement Framework (PMF) at the EMS level. The primary aim of this study was to develop a set of KPIs tailored for performance measurement of EMS in ambulance transfer cases resulting in patient collapse and to evaluate the feasibility and validity of these indicators. It is expected that if there are specific indicators for performance measurement in CIA cases; planning, and implementing interventions to improve the quality of services will be more effective.

Following the pilot testing for a year and retrospective analysis, our findings demonstrate expansion in EMS operations as evident from an increase in district/population coverage, fleet, and health workforce. It is worth highlighting that despite expansions, the incidence of CIA reduced by 7% suggesting that performance measurement activities in prehospital care have the potential to improve the system’s capacity to manage critical patient transfers as evidenced by previous literature [13]. The findings also highlighted a slight increase in average response time, attributed to expanded service delivery, and coverage areas potentially leading to delays in reaching patients. Some level of operation and procedural gaps were also identified that could have led to delays in ambulance response including gaps in communication, adherence to SOPs, and equipment management, however, exact figures cannot be elucidated based on organizational privacy and ethical limitations.

Another considerable element critical for EMS organizations is the workforce training on ethical decision-making to provide maximum benefit to the patients. In this context, an experimental study with paramedical staff demonstrated improved decision-making skills, patient-centered care, and professional gains following the workshops focused on bioethical principles derived from Islamic preachings [14]. These findings further emphasize the need to integrate ethical education as the EMS workforce is exposed to a high-pressure situation where immediate and ethical judgments are crucial to sustain and enhance EMS delivery and patient safety.

Evidence suggests that high-quality localized data is therefore vital for performance measurement and sustaining the capacity to identify, evaluate, and address system deficiencies, thereby, leading to allocative efficiency and management of available resources to focus on key areas of improvement [15, 16]. In the context of continued healthcare improvement at the prehospital level, only a few studies have evaluated the performance of EMS personnel mostly focused on the knowledge and skill assessment of Emergency Medical Dispatchers (EMDs), Emergency Telecommunicators (ETCs), and Emergency Medical Technicians (EMTs) [17–20]. One such initiative was made to evaluate the quality of prehospital emergency anesthesia (PHEA) through the development of KPIs and progress was measured over one year. The overall process resulted in improved practices and better management and utilization of equipment, processes, documentation, and patient care thereby improving performance in high-risk procedures [21]. Nevertheless, this was the only reported study focused on a single element of EMS, and its findings are not generalizable. Consequently, a significant gap remains in the comprehensive assessment of EMS performance and/or personnel using a systems approach [12, 22–25].

Additionally, it was also noted that earlier performance measurement was mainly limited to response time metrics owing to its ease of application; however, response time alone cannot be accounted for to compare performance and can negatively affect the morale of EMS personnel leading to ambulance crashes. Further, meeting a response time target does not indicate a better quality of prehospital care or improved patient outcomes, especially with modern prehospital care in which paramedics/EMTs are engaged in wide-ranging diagnostic and therapeutic interventions [26]. Some studies were conducted for the development of KPIs to assess the quality of specific components of EMS or performance assessment in the events of Road Traffic Injuries [27].

The purpose and findings of our study align with previous literature that highlights the significance of performance measurement using structured frameworks and the development of KPIs in the field of prehospital emergency care [21, 28, 29]. Nevertheless, existing literature often presents a significant limitation where key performance indicators (KPIs) are proposed but not empirically validated. Our study addresses this critical gap by not only developing KPIs but also rigorously pilot-testing them over a year, followed by a comprehensive retrospective analysis. Being the first of its study and efforts towards healthcare improvement in EMS, the study is subjected to a few limitations including the retrospective nature of data analysis that may have introduced biases, as it mainly relies on organizational records and hence did not capture data from other EMS centers. This also limits the generalizability, however, the adoption of PMF in other EMS settings has the potential to result in similar or advanced levels of improvement.

## Conclusions

This study marks a significant effort in designing and implementing a Performance Measurement Framework (PMF) to assess and improve the performance of EMS and prehospital care focusing on cases specifically resulting in CIA. Our findings highlight key areas of improvement within operations and addressing those deficiencies led to a reduction in the incidence of CIA cases despite service expansions. The findings underline insights into system-level weaknesses and challenges faced by EMS; thereby leading us to the development of targeted interventions. The study proposes areas requiring improvement such as training guidelines, adherence to operating protocols, and resource optimization. In addition; the integration of technology and advanced training programs for the ambulance workforce may strengthen the overall EMS performance; thereby promising positive patient outcomes, and efficient service delivery and service utilization. Future research should also involve longitudinal prospective studies across multiple centers to monitor the performance of EMS on a routine basis to sustain the process and improve patient outcomes. Additionally, exploring automated data collection systems could enhance the framework’s scalability and effectiveness.

## Data Availability

The study tools will be available upon request as they are the organization’s personalized record forms.

## Abbreviations

CIA: Collapse in Ambulance
CME: Continued Medical Education
EMD: Emergency Medical Dispatcher
EMS: Emergency Medical Services
EMT: Emergency Medical Technician
ETC: Emergency Telecommunicator
IAED: International Academy of Emergency Dispatch
IOM: Institute of Medicine
KPI: Key Performance Indicator
MEAL: Monitoring, Evaluation, Accountability, and Learning
MO: Medical Officer
OHCA: Out-of-Hospital Cardiac Arrest
PHEA: Prehospital Emergency Anesthesia
PMF: Performance Measurement Framework
RDE: Research, Development, and Education
SOP: Standard Operating Procedure
